# Value of routine test for identifying colorectal cancer from patients with nonalcoholic fatty liver disease

**DOI:** 10.1186/s12876-020-01327-7

**Published:** 2020-06-09

**Authors:** Rong Yang, Yu Chen, Xianlai Chen

**Affiliations:** 1 Xiangya Hospital, Central South University, Changsha, Hunan 410078 P.R. China; 2 Xiangya School of Medicine, Central South University, Changsha, Hunan 410013 P.R. China; 3 Institute of Information Security and Big Data, Central South University, 932 South Lushan Rd, Changsha, Hunan 410083 P.R. China; 4 National Engineering Laboratory for Medical Big Data Application Technology, Central South University, Changsha, Hunan 410083 P.R. China

**Keywords:** Nonalcoholic fatty liver disease, Colorectal neoplasms, Diagnostic tests, routine, Early detection of Cancer

## Abstract

**Background:**

Nonalcoholic fatty liver disease (NAFLD) is a risk factor for colorectal neoplasms. Our goal is to explore the relationship between NAFLD and colorectal cancer (CRC) and to analyze potential indicators for screening CRC in NAFLD based on clinical big data.

**Methods:**

Demographic information and routine clinical indicators were extracted from Xiangya Medical Big Data Platform. 35,610 NAFLD cases without CRC (as group NAFLD-CRC), 306 NAFLD cases with CRC (as group NAFLD-NonCRC) and 10,477 CRC cases without NAFLD were selected and evaluated. The CRC incidence was compared between NAFLD population and general population by Chi-square test. Independent sample t-test was used to find differences of age, gender and routine clinical indicators in pairwise comparisons of NAFLD-CRC, NAFLD-NonCRC and nonNAFLD-CRC.

**Results:**

NAFLD population had a higher CRC incidence than general population (7.779‰ vs 3.763‰, *P* < 0.001). Average age of NAFLD-CRC (58.79 ± 12.353) or nonNAFLD-CRC (59.26 ± 13.156) was significantly higher than NAFLD-nonCRC (54.15 ± 14.167, *p* < 0.001). But age had no significant difference between NAFLD-CRC and nonNAFLD-CRC (*P* > 0.05). There was no different gender distribution for three groups (P > 0.05). NAFLD-CRC had lower anaemia-related routine clinical indicators such as decrease of red blood cell count, mean hemoglobin content and hemoglobin than NAFLD-nonCRC (*P* < 0.05 for all). Anemia of NAFLD-CRC was typical but it might be slighter than nonNAFLD-CRC. More interestingly, NAFLD-CRC had distinct characteristics of leukocyte system such as lower white blood cell count (WBC) and neutrophil count (NEU_C) and higher basophil percentage (BAS_Per) than nonNAFLD-CRC and NAFLD-nonCRC (*P* < 0.05 for all). Compared with NAFLD-nonCRC, the change of WBC, BAS_Per and NEU_C in NAFLD-CRC was different from that in nonNAFLD-CRC. In addition, NAFLD-CRC had a higher level of low density lipoprotein (LDL) and high density lipoprotein (HDL), lower level of triglyceride (TG) and Albumin-to-globulin ratio (A/G) than NFLD-nonCRC (*P* < 0.05 for all).

**Conclusions:**

NAFLD is associated with a high incidence of CRC. Age is an important factor for CRC and the CRC incidence increases with age. Anemia-related blood routine clinical indicators, leukocyte system and blood lipid indicators may be more important variables for identifying CRC in NAFLD. So blood routine test and liver function/blood lipid test are valuable for screening CRC in NAFLD.

## Background

Colorectal cancer (CRC) is the third most common cancer in men and the second one in women worldwide in 2012 [1]. It is estimated as the second cause of cancer death and new cases in the United States in 2018 [2]. Early screening, timely diagnosis and surgical treatment of CRC can improve the prognosis of patients and reduce the mortality by 15–30% [3]. Such screening approaches as fecal occult blood test and colonoscopy have played an active role on reducing the incidence and mortality of CRC in both observational and controlled trials [4, 5]. According to the guidelines for CRC screening issued by the World Health Organization, it is recommended that people aged 50–70 years or over need regular CRC screening [6]. However, patients might pay their attentions to it only when symptoms have appeared, such as hematochezia, frequent stools, lower abdominal pain, multiple diarrhea and so on. Unfortunately, many symptoms manifest only in the terminal stage of CRC so patients with early cancer often ignore or fail to report these symptoms. In addition, some patients are reluctant to do CRC screening due to self-perceived health or physical/psychological barriers, which will delay timely diagnosis and treatment and result in serious consequences. Therefore, exploring the risk factors and valuable diagnostic indicators related to malignant colorectal tumors will be helpful for improving early detection of CRC.

It has been found in epidemiological studies that diabetes mellitus, obesity and metabolic syndrome are related to CRC [7, 8]. Non-alcoholic fatty liver disease (NAFLD), the second most common liver disease after viral hepatitis, also has a correlation with diabetes, obesity and metabolic syndrome. It affects 20–40% of adults and may have potential to evolve to non-alcoholic steatohepatitis, cirrhosis or liver cancer [9, 10]. There are many common risk factors for NAFLD and CRC, such as metabolic syndrome, obesity and insulin tolerance. It is shown that the CRC incidence in NAFLD patients is higher [11]. In this paper, based on the clinical data, the risk factors and diagnostic indicators of CRC in NAFLD will be explored to help detect early CRC in NAFLD population by routine clinical test.

## Methods

### Subjects

From Xiangya Medical Big Data Platform, which integrates medical data derived from Xiangya Hospital, the Second Xiangya Hospital and the Third Xiangya Hospital of Central South University, the inpatient cases from April 2002 to November 2015 were collected. Inclusion criteria: NAFLD was clearly diagnosed and CRC was determined by pathological finding. Cases with recent history of colorectal polyps, asymptomatic enteritis, other tumors, viral hepatitis, alcoholic liver disease, excessive drinking or acute fatty liver of pregnancy were excluded. In this study, a total of 35,916 NAFLD patients met the inclusion criteria, including 306 cases with CRC (as group NAFLD-CRC) and 35,610 cases without CRC (as group NAFLD-NonCRC). In addition, 10,477 CRC cases without NAFLD were also collected as group nonNAFLD-CRC.

### Collecting & processing data

Referring to the relevant literature and considering universality, cost and convenience of the existing detection methods, some candidate factors and indicators were extracted from the data set. It involves blood routine, liver function/blood lipid and demographic information. Blood routine includes 22 indicators such as white blood cell count (WBC), monocyte percentage (MONO_PER), monocyte count (MONO_C), neutrophil percentage (NEU_PER), neutrophil count (NEU_C), basophil percentage (BAS_PER), basophil count (BAS_C), eosinophil percentage (EOS_PER), eosinophil count (EOS_C), lymphocyte percentage (LYMPH_PER), lymphocyte count (LYMPH_C), hematocrit (HCT), red blood cell count (RBC), red blood cell volume distribution width (RDW), mean corpuscular volume (MCV), mean hemoglobin content (MCH), mean corpuscular hemoglobin concentration (MCHC), hemoglobin (Hb), platelet (PLT), mean platelet volume (MPV), platelet volume distribution width (PDW) and plateletcrit (PCT). 11 indicators of liver function/blood lipid were extracted, such as alanine aminotransferase (ALT), aspartate aminotransferase (AST), thrombin time (TT), prothrombin time (PT), total protein (TP), albumin (ALB), globulin (GLB), albumin-to-globulin ratio (A/G), low density lipoprotein (LDL), high density lipoprotein (HDL) and triglyceride (TG). Demographic information consisted of age and gender. Except for gender as a categorized variable, other variables were treated as continuous data. Age was processed and analyzed as the continuous value as well as it was divided into age groups of 0–17, 18–39, 40–49, 50–65 and 66 years or over referring to the age group criteria in China and considering the study results of literature [12]. Age distribution of subjects was analyzed by group.

### Incidence analysis of CRC in NAFLD population

In order to explore the difference of CRC between NAFLD population and general population, the CRC incidence in the two populations were compared and analyzed. The CRC incidence in the general population was estimated by “Cancer Statistics of China 2015” [13]. The CRC incidence in NAFLD population was calculated according to the data collected in this study. Then, chi square test was used to find the difference of CRC incidence between the two populations. In addition, the CRC incidence of NAFLD population was analyzed by age stage.

### Correlation analysis between factors or indicators and CRC in NAFLD patients

Statistical methods were used to compare NAFLD-CRC, NAFLD-NonCRC and nonNAFLD-CRC in order to find the differences of variables among the three groups and reveal the relationship between the variables and CRC. The categorical variables were expressed as percentage and then compared by chi square test. Gender was treated as a categorical variable. Other continuous variables were analyzed by independent sample t-test. Those continuous variables included age, WBC, MONO_PER, MONO_C, HCT, RBC, RDW, LYMPH_PER, LYMPH_C, MCV, MCH, MCHC, BAS_PER, BAS_C, EOS_PER, EOS_C, Hb, PLT, MPV, PCT, NEU_C, PDW, NEU_PER, ALB, TP, GLB, A/G, LDL, HDL, TG, ALT, AST, PT and TT.

## Results

### CRC incidence in NAFLD population

In order to evaluate the CRC incidence in NAFLD population, general population was used to compare. According to “China Cancer Statistics 2015” [13], the CRC incidence in general population in China was estimated about 3.763‰ in 2015. In this study, 306 of 35,916 NAFLD cases were diagnosed as CRC. Therefore, the CRC incidence in NAFLD population was about 8.520‰. The CRC incidence in NAFLD population was significantly higher than general population (*X*^2^ = 119.917, *P* = 0.000 < 0.001).

### Demographic factors and CRC in NAFLD and nonNAFLD

As shown in Table 1, there were 21,227 (59.1%) males and 14,689 (40.9%) females in 35,916 NAFLD cases. The male-to-female ratio was about 1.45: 1. NAFLD-CRC includes 167 (54.6%) males and 139 (45.4%) females. NAFLD-NonCRC consisted of 21,060 (59.1%) males and 14,550 (40.9%) females. There were 6205(59.2%) males and 4272 (40.8%) females in NonNAFLD-CRC. No significant differences of gender distribution were identified among the three groups (*X*^2^ = 2.668, *P* = 0.263 > 0.05).

**Table 1:** Gender distribution in NAFLD-CRC, nonNAFLD-CRC and NAFLD-nonCRC

Gender	NAFLD-CRC n(%)	NAFLD-NonCRC n(%)	nonNAFLD-CRC n(%)	*X*^2^	P
Male	167 (54.6)	21,060 (59.1)	6205 (59.2)	2.668	0.263
Female	139 (45.4)	14,550 (40.9)	4272 (40.8)		
total	306 (100)	35,610 (100)	10,477 (100)		

The age difference was compared by independent sample t-test. The results were shown in Table 2. The average age of NAFLD-CRC was higher than NAFLD-NonCRC (58.79 ± 12.353 vs 54.15 ± 14.167, t = − 6.536, *P* = 0.000 < 0.001). That of nonNAFLD-CRC was also higher than NAFLD-nonCRC (59.26 ± 13.156 vs 54.15 ± 14.167, t = − 34.361, P = 0.000 < 0.001). However, there had no significant difference between nonNAFLD-CRC and NAFLD-CRC (t = − 0621, *P* = 0.534 > 0.05).

**Table 2:** Age difference among NAFLD-CRC, nonNAFLD-CRC and NAFLD-nonCRC

group	$\overline{x}\pm s$	t_value	P
NAFLD-CRC vs NAFLD-NonCRC	58.79 ± 12.353 vs 54.15 ± 14.167	−6.536	0.000
NAFLD-CRC vs nonNAFLD-CRC	58.79 ± 12.353 vs 59.26 ± 13.156	−0.621	0.534
NAFLD-NonCRC vs nonNAFLD-CRC	54.15 ± 14.167 vs 59.26 ± 13.156	−34.361	0.000

According to the age group criteria in China, age was divided into following stages: minor (0–17 years), young (18–40 years), middle-aged (41–65 years) and old (66 years or above). In literature [12], it was found that the CRC incidence in 40–49 years population was also relatively high. So the cases in our study were divided into five age groups: 0–17 years, 18–39 years, 40–49 years, 50–65 years and 66 years or over. The distribution of CRC in NAFLD population by age was shown in Table 3. There are 209 NAFLD-NonCRC cases and no NAFLD-CRC case in the 0–17 years group, 4857 NAFLD-NonCRC cases and 16 NAFLD-CRC cases with CRC ratio of 3.283‰ in the 18–39 years group, 8845 NAFLD-NonCRC cases and 72 NAFLD-CRC cases with CRC ratio of 8.074‰ in the 40–49 years group, 14,047 NAFLD-NonCRC cases and 117 NAFLD-CRC cases with CRC ratio of 8.260‰ in the 50–65 years group and 7652 NAFLD-NonCRC cases and 101 NAFLD-CRC cases with CRC ratio of 13.027‰ in the 66 years or over group. Percentages of age group 0–17, 18–39, 40–49, 50–65 and 66 years or over were respectively 0, 5.23, 23.53, 38.24 and 33.01% in NAFLD-CRC, respectively 0.11, 6.82, 17.13, 42.06 and 33.86% in nonNAFLD-CRC.

**Table 3:** Age distribution of cases in NAFLD-NonCRC, NAFLD-CRC and nonNAFLD-CRC

Age group	NAFLD-CRC #(%), a	NAFLD-NonCRC #(%), b	nonNAFLD-CRC #(%)	CRC ratio, a/(a + b)
0–17	0 (0)	209 (0.59)	12 (0.11)	0
18–39	16 (5.23)	4857 (13.64)	715 (6.82)	3.283‰
40–49	72 (23.53)	8845 (24.84)	1795 (17.13)	8.074‰
50–65	117 (38.24)	14,047 (39.45)	4407 (42.06)	8.260‰
> = 66	101 (33.01)	7652 (21.49)	3548 (33.86)	13.027‰
total	306 (100)	35,610 (100)	10,477 (100)	8.520‰

### Blood routine test and CRC in NAFLD and nonNAFLD

Blood routine clinical indicators were evaluated by independent sample t-test in pairwise comparisons of NAFLD-nonCRC, NALFD-CRC and nonNAFLD-CRC. The results were shown in Table 4. MONO_C, LYMPH_PER, BAS_C, EOS_PER, EOS_C, NEU_PER, RDW were not significantly different between NAFLD-nonCRC and NAFLD-CRC (*P* < 0.05 for all). However, NAFLD-CRC had significantly higher values of MONO_PER, BAS_PER, PCT and PLT and lower values of WBC, LYMPH_C, NEU_C, HCT, RBC, MCV, MCH, MCHC, Hb, MPV, PDW and PCT when compared with the NAFLD-nonCRC (P < 0.05 for all). All blood routine clinical indicators were significantly different between NAFLD-nonCRC and nonNAFLD-CRC (P < 0.05 for all). Compared with the NAFLD-nonCRC, nonNAFLD-CRC had significantly lower values of LYMPH_PER, LYMPH_C, BAS_PER, BAS_C, HCT, RBC, MCV, MCH, MCHC, Hb, MPV and PDW and higher values of WBC, MONO_PER, MONO_C, EOS_PER, EOS_C, NEU_PER, NEU_C, RDW, PCT and PLT(*P* < 0.001 for all). Between NAFLD-CRC and nonNAFLD-CRC, MONO_PER, MCV, MPV and PCT were not significantly different (*P* > 0.05 for all). But NAFLD-CRC have significantly lower values of WBC, MONO_C, BAS_C, EOS_PER, EOS_C, NEU_PER, NEU_C, RDW and PLT and higher values of LYMPH_PER, LYMPH_C, BAS_PER, HCT, RBC, MCH, MCHC, Hb and PDW when compared with the nonNAFLD-CRC (*P* < 0.05 for all).

**Table 4:** comparison of blood routine clinical indicators among NAFLD-NonCRC, NAFLD-CRC and nonNAFLD-CRC

Indicator	$\overline{x}\pm s(n)$			P		
	NAFLD_nonCRC (G_1_)	NAFLD_CRC (G_2_)	nonNAFLD-CRC (G_3_)	G_1_ vs G_2_	G_1_ vs G_3_	G_2_ vs G_3_
WBC(10^9^/L)	7.63 ± 3.46 (31598)	6.86 ± 2.27 (279)	7.98 ± 3.91 (4607)	0.000	0.000	0.000
MONO_PER(%)	6.27 ± 2.61 (31290)	6.69 ± 2.3 (279)	6.56 ± 2.73 (10415)	0.008	0.000	0.452
MONO_C(10^9^/L)	0.47 ± 0.56 (31268)	0.45 ± 0.2 (279)	0.49 ± 0.27 (10414)	0.477	0.000	0.001
LYMPH_PER(%)	26.21 ± 10.42 (31613)	26 ± 9.84 (279)	18.64 ± 9.83 (10417)	0.733	0.000	0.000
LYMPH_C(10^9^/L)	1.81 ± 0.77 (31594)	1.68 ± 0.61 (279)	1.26 ± 0.63 (10416)	0.000	0.000	0.000
BAS_PER(%)	0.45 ± 0.39 (31273)	0.5 ± 0.35 (279)	0.39 ± 0.37 (10147)	0.029	0.000	0.000
BAS_C(10^9^/L)	0.03 ± 0.04 (31267)	0.03 ± 0.03 (279)	0.03 ± 0.04 (9172)	0.949	0.000	0.011
EOS_PER (%)	2.29 ± 2.33 (31277)	2.38 ± 1.92 (279)	2.88 ± 2.63 (10330)	0.505	0.000	0.000
EOS_C(10^9^/L)	0.16 ± 0.23 (31260)	0.16 ± 0.14 (279)	0.2 ± 0.19 (10259)	0.990	0.000	0.000
NEU_PER(%)	64.3 ± 11.87 (31607)	64.15 ± 11.13 (279)	71.1 ± 11.85 (10416)	0.829	0.000	0.000
NEU_C(10^9^/L)	5.12 ± 3.18 (31587)	4.52 ± 2.13 (279)	5.76 ± 3.52 (10416)	0.000	0.000	0.000
HCT(%)	40.16 ± 5.96 (31608)	38.16 ± 6.41 (279)	33.5 ± 5.84 (10416)	0.000	0.000	0.000
RBC(10^12^/L)	4.44 ± 0.7 (31609)	4.33 ± 0.77 (279)	3.97 ± 0.64 (4611)	0.026	0.000	0.000
RDW(%)	13.97 ± 4.32 (31417)	14.34 ± 2.4 (278)	14.79 ± 3.2 (7114)	0.159	0.000	0.003
MCV (fl)	90.89 ± 6.78 (31614)	88.59 ± 9.27 (279)	88.29 ± 8.37 (10417)	0.000	0.000	0.551
MCH (pg)	30.18 ± 2.56 (31615)	29 ± 3.75 (279)	28.47 ± 3.43 (10417)	0.000	0.000	0.012
MCHC(g/L)	331.17 ± 16.92 (27082)	326.07 ± 19.06 (258)	321.96 ± 18.92 (10417)	0.000	0.000	0.001
Hb(g/L)	133.45 ± 20.82 (31609)	124.9 ± 23.18 (279)	108.13 ± 20.82 (10416)	0.000	0.000	0.000
PLT(10^9^/L)	199.41 ± 71.34 (31609)	228.12 ± 81.52 (279)	251.11 ± 111.17 (10416)	0.000	0.000	0.000
MPV (fl)	10.63 ± 1.66 (30514)	10.24 ± 1.63 (267)	10.22 ± 2.98 (10328)	0.000	0.000	0.943
PDW(%)	15.39 ± 2.64 (24421)	14.85 ± 2.54 (240)	14 ± 2.71 (6692)	0.002	0.000	0.000
PCT(%)	0.22 ± 0.07 (24774)	0.23 ± 0.08 (247)	0.31 ± 3 (8793)	0.001	0.003	0.683

### Liver function/blood lipid and CRC in NAFLD and nonNAFLD

Liver function/blood lipid indicators were analyzed by independent sample t-test in pairwise comparisons of NAFLD-CRC, NAFLD-NonCRC and nonNAFLD-CRC. The results were shown in Table 5. It could be found that LDL and HDL were higher (*P* < 0.05 for both), while TG and A/G (P < 0.05 for both) were lower in NAFLD-CRC than those in NAFLD-NonCRC. Other liver function/blood lipid clinical indicators had no significant difference between the two groups (*P* > 0.05 for all). All liver function/blood lipid clinical indicators were significantly different between NAFLD-nonCRC and nonNAFLD-CRC. NAFLD-nonCRC had higher values of ALT, AST, TT, TP, ALB, GLB, A/G, LDL and TG and lower value of PT and HDL than nonNAFLD-CRC (*P* < 0.001 for all). Compared with NAFLD-CRC, nonNAFLD-CRC had higher values of ALT, TP, ALB, GLB, A/G, LDL, TT and TG (*P* < 0.05 for all) and lower value of PT (P < 0.001). AST and HDL were not significantly different between NAFLD-CRC and nonNAFLD-CRC.

**Table 5:** comparison of liver function/blood lipid indicators among NAFLD-NonCRC, NAFLD-CRC and nonNAFLD-CRC

Indicator	$\overline{x}\pm s(n)$			P		
	NAFLD_nonCRC (G1)	NAFLD_CRC (G_2_)	nonNAFLD-CRC (G_3_)	G_1_ vs G_2_	G_1_ vs G_3_	G_2_ vs G_3_
ALT(U/L)	39.16 ± 71.79 (15850)	29.47 ± 34.4 (108)	22.44 ± 36.69 (8433)	0.161	0.000	0.048
AST(U/L)	33.48 ± 87.36 (15532)	29.67 ± 54.8 (107)	25.86 ± 31.84 (8384)	0.653	0.000	0.224
TP(g/L)	67.17 ± 7.85 (30654)	66.97 ± 8.26 (273)	62.33 ± 7.38 (10263)	0.688	0.000	0.000
ALB(g/L)	40.49 ± 5.67 (30731)	40.19 ± 6.93 (273)	36.04 ± 5.5 (10324)	0.479	0.000	0.000
GLB(g/L)	26.77 ± 5.19 (30629)	27.17 ± 5.17 (273)	26.32 ± 4.9 (10260)	0.199	0.000	0.005
A/G	1.58 ± 0.34 (22965)	1.53 ± 0.35 (231)	1.42 ± 0.35 (10259)	0.036	0.000	0.000
LDL (mmol/L)	2.79 ± 1.09 (27366)	3.04 ± 0.97 (224)	2.58 ± 0.9 (4279)	0.001	0.000	0.000
HDL (mmol/L)	1.13 ± 0.42 (27418)	1.2 ± 0.38 (224)	1.19 ± 0.35 (2916)	0.010	0.000	0.707
TT(s)	16.8 ± 4.85 (17357)	16.65 ± 3.6 (245)	16.18 ± 3.26 (8648)	0.627	0.000	0.042
PT(s)	12.21 ± 2.09 (17452)	11.95 ± 1.31 (244)	12.5 ± 2.4 (8616)	0.059	0.000	0.000
TG (mmol/L)	2.49 ± 2.75 (28567)	1.74 ± 0.94 (236)	1.3 ± 0.83 (4365)	0.000	0.000	0.000

## Discussion

### NAFLD and CRC

In our study, it is found that the CRC incidence of NAFLD population is significantly higher than that of the general population (8.520‰ vs 3.763‰, P < 0.001). It is suggested that NAFLD population has a higher risk of CRC, which is consistent with some previous studies. For example, Kim GA [14] suggested that NAFLD was associated with colorectal cancer development in males. Their study showed that NAFLD had a high score of fibrosis and fibrosis-4 and it was a strong association with the development of all cancers and hepatocellular carcinoma. In a study on relationship between NAFLD and malignant colorectal neoplasms (CRMN), Lin XF [15] also found that the CRC incidence in NAFLD population was significantly higher than control group. There is a significant correlation between NAFLD and CRMN. NAFLD was considered as an independent risk factor for CRMN. Pan S [16] investigated the relationship between colorectal tumors and NAFLD, metabolic syndrome (MetS). They believed that NAFLD and MetS were risk factors for CRC and had a collateral effect on the development of CRC. Meta-analysis by Mantovani A [17] showed that NAFLD was independently associated with CRC. The mechanism between NAFLD and CRC is not clear, but NAFLD represents severe insulin resistance (IR) and inflammatory response. Insulin and insulin-like growth factor may promote the development of CRC through proliferation and apoptosis [18]. Many factors affect the cancerization progression of NAFLD. IR, chronic inflammation, allergy and adipose tissue disorders play a key role in the progression of extrahepatic tumors in NAFLD population [19]. Meanwhile, a new research found that gut microbiota abnormalities occurred in NAFLD patients [20]. Another research showed that bacterial metabolism of bile acids could promote generation of peripheral regulatory T cells which regulate intestinal inflammation [21]. Chronic inflammation has something to do with CRC. So it’s possible that gut microbiota abnormalities appearing in NAFLD patients may induce the development of CRC or NAFLD may disturb the distribution of gut bacteria, finally promoting CRC. In a word, NAFLD is closely related to CRC and it is an important factor in the development of CRC. NAFLD individual should be paid more attention to the risk of CRC than general population.

### Correlation between gender, age and CRC in NAFLD population

The results (Table 1) show that there are 167 males (54.6%) vs 139 females (45.4%) in NAFLD-CRC, 21060 males (59.1%) vs 14,550 females (40.9%) in NAFLD-nonCRC and 6205 males (59.2%) vs 4272 females (40.8%) in nonNAFLD-CRC. After analysis, it reveals that gender distribution will not influence the incidence of CRC in NAFLD population, although male patients are more than female patients in each group (*P* > 0.05). Study from Pan S [16] also suggested that there was no significant difference for gender distribution between NAFLD with CRC and NAFLD without CRC. So it can be inferred that gender is not a key factor related to CRC in NAFLD population.

From analysis result by age (Table 2), it can be found that the average age of NAFLD-CRC (58.79 ± 12.353) or nonNAFLD-CRC (59.26 ± 13.156) is higher than that of NAFLD-NonCRC (54.15 ± 14.167, *P* < 0.001). But there is no significant age difference between NAFLD-CRC and nonNAFLD-CRC (*P* > 0.05). Table 3 shows that with the increase of age, the percentage of cases becomes higher and higher in NAFLD-CRC or nonNAFLD-CRC and the CRC incidence also increases in NAFLD population. Group 66 years or over has the highest CRC ratio (13.027‰), which is nearly four times as that of group 18–39 years (3.283‰). The CRC ratio of group 40–49 years (8.074) or group 50–65 years (8.260‰) is more than twice as that of group 18–39 years (3.283‰). But CRC case is not found in the underage (0–17 years). The results suggest that the CRC incidence in NAFLD population has a strong correlation with age. It is in line with the general rule that tumors occur more frequently in the elderly population. Therefore, clinical guidelines recommend colonoscopy for early detection of colorectal tumors in people aged 50–70 or older [22]. In addition, Chen CH [23] suggested that the screening age for CRC should be reduced to 40 years old in order to early detect it. Our study also shows that the CRC incidence of group 40–49 years rises sharply up in the NAFLD population. So age should be used as one of the important factors of CRC screening.

### Value of blood routine test for predicting CRC in NAFLD population

Jellema P [24] considered that diagnostic performance of blood test for CRC is limited in clinical practice when used as a single test but anemia is useful symptoms for CRC detection. Our results (Table 4) show that HCT, RBC, MCV, MCH, MCHC, Hb, MPV and PDW of NAFLD-CRC and nonNAFLD-CRC are lower, PLT and PCT are higher, than those of NAFLD-NonCRC (*P* < 0.05 for all). It implies that CRC has a higher possibility of anemia. However, NAFLD-CRC has higher values of HCT, RBC, MCH, MCHC and Hb (P < 0.05 for all) and lower values of RDW, PLT and PDW (P < 0.05 for all), compared with nonNAFD-CRC. Anemia in NAFLD-CRC may not be serious as nonNAFLD-CRC. The decrease or increase of indicators related to blood cell and hemoglobin may result from occult intestinal bleeding due to CRC. Combined with fecal occult blood test, anemia-related blood indicators may be a valuable tool for CRC screening in NAFLD population.

Kato M [25] suggested that the decrease of MCV could be used as an independent predictor of late CRC and further colonoscopy was necessary for people over 85 years with the decrease of MCV. In our study, the MCV of NAFLD-CRC (88.59 ± 9.27 fl) and nonNAFLD-CRC (88.29 ± 8.37 fl) is significantly lower than that of NAFLD-NonCRC (90.89 ± 6.78 fl, *P* < 0.001). The results confirm that the decrease of MCV in NAFLD patients may be related to CRC.

Malignant tumors are often accompanied by elevated platelets. Platelets can aggregate and degranulate in tumor microvessels, and release platelet transformation and derivation growth factors, thus stimulating the growth of tumor cells. On the other hand, thrombopoietin-like hormone produced by cancer cells and tumor-related inflammatory mediators can also stimulate platelet elevation [26]. So the number of platelet may rise up in cancer patients. Table 4 shows that the platelet of NAFLD-CRC (228.12 ± 81.52*10^9^/L) and nonNAFLD-CRC (251.11 ± 111.17*10^9^/L) are higher than that of NAFLD-NonCRC (199.41 ± 71.34*10^9^/L, *P* < 0.001). It indicates that the number of platelets is valuable for screening CRC of NAFLD population.

The study [27] has shown that serum PCT level is a fast and reliable laboratory indicator for early diagnosis of infectious complications after operation for colorectal cancer. Our study results (Table 4) show that PCT of NAFLD-CRC (0.23 ± 0.08%) and nonNAFLD-CRC (0.31 ± 3%) are significantly higher than that of NAFLD-NonCRC (0.22 ± 0.07%, *P* < 0.05), which indicates possible value of PCT in identifying CRC patients in NAFLD population.

In the case of cancer, leukocyte system may change. Evani SJ [28] found that Tumor-Associated Macrophage (TAM) derived from monocytes could release cytokines and angiogenic factors and promote the progress and metastasis of tumors. Chanmee T [29] also found that TAM could secrete a large number of angiogenic factors, thereby promoting tumor angiogenesis. So an increase in the percentage of peripheral blood monocytes may be associated with tumors. Our results (Table 4) show that NAFLD-CRC and nonNAFLD-CRC have a higher MONO_PER and lower LYMPH_C than NAFLD-NonCRC (*P* < 0.05 for both). However, WBC of NAFLD-CRC is significantly lower than nonNAFLD-CRC and NAFLD-nonCRC (*P* < 0.001 for both). More interestingly, the changes of WBC, BASO_Per and NEU_C in NAFLD-CRC are apparently different from those in nonNAFLD-CRC. Compared with NAFLD-nonCRC, NAFLD-CRC has lower WBC and NEU_C and higher BASO_PER. But nonNAFLD-CRC has higher WBC and NEU_C and lower BASO_PER than NAFLD-nonCRC. Decreasing WBC and NEU_C, increasing BASO_PER are important features for leukocyte system in NAFLD-CRC differing from nonNAFLD-CRC. Leukocyte system change of NAFLD-CRC is different from NAFLD-nonCRC and nonNAFLD-CRC. It is suggested that the change of peripheral blood leukocyte system may be valuable for screening CRC in NALFD population.

In summary, it can be inferred that both NAFLD-CRC and nonNAFLD-CRC may have anaemia symptom and occur leukocyte system change. But anaemia of NAFLD-CRC may be slighter than nonNAFLD-CRC. The change of leukocyte system of NAFLD-CRC, especially WBC, BASO_PER and NEU_C, may be different from nonNAFLD-CRC. So routine blood test is valuable for screening CRC from NAFLD population.

### Value of liver function/blood lipid for CRC prediction in NAFLD population

As shown in Table 5, except for AST and HDL with no significant difference (*P* > 0.05 for both), NAFLD-CRC have a lower level of PT (*P* < 0.001) and a higher level of other liver function/blood lipid indicators (*P* < 0.05 for all) than nonNAFLD-CRC. There are significant differences of such indicators as HDL, LDL, A/G and TG between NAFLD-CRC and NAFLD-NonCRC (P < 0.05 for all). But no significant correlation has been found for other liver function/blood lipid indicators, including ALB, ALT, AST, TP, GLB, PT and TT (P > 0.05 for all). It is shown that there has significant difference of liver function/blood lipid between NAFLD and non NAFLD. These results suggest that HDL, LDL, A/G and TG may be the valuable indicators for identifying CRC from NAFLD population.

In a large cross-sectional study, Yang [30] found that the increase of HDL level was related to the increase of the incidence of colorectal benign tumors. However, Park [31] suggested that low HDL level was an independent risk factor for advanced colorectal cancer. Chandler [32] also found that HDL was inversely correlated with CRC risk. Zhang [33] and Tian [34] suggested that there was no significant correlation between HDL level and the incidence of colorectal tumors. Our results show that HDL of NAFLD-CRC and nonNAFLD-CRC is significantly higher than that of NAFLD-nonCRC (*P* < 0.05 for both). It is suggested that HDL may increase in the case of CRC. Zhang [29] found that LDL was significantly lower in CRC patients, compared with benign colorectal cancer patients and healthy people. But Tian [34] pointed out that the increased LDL level was related to the increased incidence of colorectal tumors. Our results show that LDL of NAFLD-CRC is higher than that of NAFLD-nonCRC and nonNAFLD-CRC (P < =0.001 for both). It reveals that a high level of LDL level may be related to NAFLD-CRC.

Yang [27] and Chandler [29] considered that the high level of TG was positively related to CRC risk. Our study finds that TG of NAFLD-CRC and nonNAFLD-CRC is lower than that of NALFD-nonCRC (*P* < 0.001 for both). But NAFLD-CRC has a higher level of TG than nonNAFLD-CRC (P < 0.001). It may be inferred that decrease of TG be correlated with CRC in population. NAFLD individual should be paid more attention to the rapid change of TG.

In addition, our results show that A/G level of NAFLD-CRC and nonNALFD-CRC is lower than that of NAFLD-nonCRC (*P* < 0.05 for both). It implies that decrease of A/G may be useful for detection of CRC in NALFD population.

The above results suggest that HDL, LDL, TG and A/G may be important indicators for CRC in NAFLD population. Abnormal value of HDL, LDL, TG point to abnormal lipid metabolism. On the one hand, Imbalances between liver lipid output and input are the direct causes of NAFLD [35]. The fact that NAFLD patients always have obesity may explain the increment of HDL, LDL, TG. On the other hand, lipid metabolism reprogramming occurs in CRC patients’ cancer-associated fibroblasts, mainly in the increase of fatty acids, phospholipids, and glycerides [36]. The decrease of A/G relates to inflammation. We still need more evidence to explore whether there exists correlations between them. The change of HDL, LDL, TG and A/G can be considered as indicators for screening CRC in NAFLD population.

## Conclusions

The CRC incidence in NAFLD population is higher than that in general population. The age is an important factor for CRC and the CRC incidence increases with age. The CRC incidence of male is higher than that of female, but gender distribution difference is not found among NAFLD-CRC, NAFLD-nonCRC and nonNAFLD-CRC. Some routine clinical indicators are significantly different between NAFLD-CRC or nonNAFLD-CRC and NAFLD-NonCRC. Anaemia and the change of peripheral blood leukocyte system may be related to CRC in NALFD population. But anaemia of NAFLD-CRC may be slighter than nonNAFLD-CRC and leukocyte system change of the former may be different from the latter, especially WBC, BASO_PER and NEU_C. HDL, LDL, TG and A/G may be useful indicators for screening CRC in NALFD population. So routine blood test, liver function/blood lipid test are valuable for screening CRC in NAFLD population.

## Data Availability

The datasets used and/or analyzed during the current study are available from the corresponding author on reasonable request.

## Abbreviations

A/G: Albumin-to-globulin ratio
ALB: Albumin
ALT: Alanine aminotransferase
AST: Aspartate aminotransferase
BAS_C: Basophil count
BAS_PER: Basophil percentage
CRC: Colorectal Cancer
EOS_C: Eosinophil count
EOS_PER: Eosinophil percentage
GLB: Globulin
Hb: Hemoglobin
HCT: Hematocrit
HDL: High density lipoprotein
LDL: Low density lipoprotein
LYMPH_C: Lymphocyte count
LYMPH_PER: Lymphocyte percentage
MCH: Mean hemoglobin content
MCHC: Mean corpuscular hemoglobin concentration
MCV: Mean corpuscular volume
MONO_C: Monocyte count
MONO_PER: Monocyte percentage
MPV: Mean platelet volume
NAFLD: Nonalcoholic fatty liver disease
NAFLD-CRC: NAFLD group with CRC
NAFLD-NonCRC: NAFLD group without CRC
NEU_C: Neutrophil count
NEU_PER: Neutrophil percentage
nonNAFLD-CRC: CRC without NAFLD
PCT: Plateletcrit
PDW: Platelet volume distribution width
PLT: Platelet
PT: Prothrombin time
RBC: Red blood cell count
RDW: Red blood cell volume distribution width
TG: Triglyceride
TP: Total protein
TT: Thrombin time
WBC: White blood cell count
